# The Brain-Gut-Microbiome System: Pathways and Implications for Autism Spectrum Disorder

**DOI:** 10.3390/nu13124497

**Published:** 2021-12-16

**Authors:** Michelle A. Chernikova, Genesis D. Flores, Emily Kilroy, Jennifer S. Labus, Emeran A. Mayer, Lisa Aziz-Zadeh

**Affiliations:** 1 USC Chan Division of Occupational Science and Occupational Therapy, University of Southern California, Los Angeles, CA 90033, USA; mchernik@lion.lmu.edu (M.A.C.); gdflores@cpp.edu (G.D.F.); ekilroy@usc.edu (E.K.); 2 Brain and Creativity Institute, University of Southern California, Los Angeles, CA 90089, USA; 3 Psychology Department, Loyola Marymount University, Los Angeles, CA 90045, USA; 4 Psychology Department, California State Polytechnic University, Pomona, CA 91768, USA; 5 G. Oppenheimer Center for Neurobiology of Stress and Resilience, University of California Los Angeles, Los Angeles, CA 90095, USA; jlabus@ucla.edu; 6 Vatche and Tamar Manoukian Division of Digestive Diseases, University of California Los Angeles, Los Angeles, CA 90095, USA; 7 David Geffen School of Medicine, University of California Los Angeles, Los Angeles, CA 90095, USA; 8 Gonda (Goldschmied) Neuroscience and Genetics Research Center, Brain Research Institute UCLA, Los Angeles, CA 90095, USA

**Keywords:** autism spectrum disorder, brain-gut-microbiome system, gut-brain axis, microbiome, probiotics, tryptophan pathway

## Abstract

Gastrointestinal dysfunction is one of the most prevalent physiological symptoms of autism spectrum disorder (ASD). A growing body of largely preclinical research suggests that dysbiotic gut microbiota may modulate brain function and social behavior, yet little is known about the mechanisms that underlie these relationships and how they may influence the pathogenesis or severity of ASD. While various genetic and environmental risk factors have been implicated in ASD, this review aims to provide an overview of studies elucidating the mechanisms by which gut microbiota, associated metabolites, and the brain interact to influence behavior and ASD development, in at least a subgroup of individuals with gastrointestinal problems. Specifically, we review the brain-gut-microbiome system and discuss findings from current animal and human studies as they relate to social-behavioral and neurological impairments in ASD, microbiota-targeted therapies (i.e., probiotics, fecal microbiota transplantation) in ASD, and how microbiota may influence the brain at molecular, structural, and functional levels, with a particular interest in social and emotion-related brain networks. A deeper understanding of microbiome-brain-behavior interactions has the potential to inform new therapies aimed at modulating this system and alleviating both behavioral and physiological symptomatology in individuals with ASD.

## 1. Introduction

Autism spectrum disorder (ASD) is a complex neurodevelopmental disorder characterized by two core deficits: persistent difficulties in social communication and interaction, and restricted, repetitive patterns of behavior [1]. A growing body of research suggests that gut microbiota may serve an important role in modulating brain function, social behavior, and ASD symptomatology; for a review, see [2]. While various genetic and environmental risk factors have been implicated in ASD, this review aims to analyze the putative mechanisms underlying how gut microbiota, associated metabolites, and the brain interact to influence behavior and ASD development. In particular, we review: (1) the brain-gut-microbiome (BGM) system; (2) findings from current animal and human studies as they relate to behavioral and neurological impairments in ASD; (3) the potential for microbiota-targeted therapies in ASD; and (4) how alterations to the microbiome may influence the development of neural networks involved in social and emotional skills.

## 2. Strategies for Article Search

Articles were obtained from PubMed, SCOPUS, PsycInfo, and Google Scholar databases, using the following keywords: gut microbiome, gut-brain-microbiome axis, autism, autism spectrum disorder, ASD, social behavior, social cognition, emotion processing, probiotics, fecal microbial transplantation, tryptophan metabolism, or neuroimaging. Additional articles were found using the reference lists of already retrieved studies. Our search was limited to studies written in English and published up to January 2022. Both original research and review articles were included.

## 3. The Brain-Gut-Microbiome (BGM) System

Over the past decade, a surge of research has emerged that investigates the bidirectional relationship between the brain and the human gut microbiome, known as the brain-gut-microbiome (BGM) system. Gut microbiota play a crucial role in the modulation of cross-talk between the gut and nervous system [3] and promote gastrointestinal (GI) homeostasis, in addition to impacting higher cognitive functions [2]. The BGM system includes the central and enteric nervous systems (CNS; ENS) and various neural, metabolic, endocrine, and immune mediators [2]. Some microbiota, as well as their molecular by-products, neuroactive metabolites, and related inflammatory mediators, can cross both the gut and blood-brain barriers, allowing transmission along the BGM system [4].

**Figure 1:**
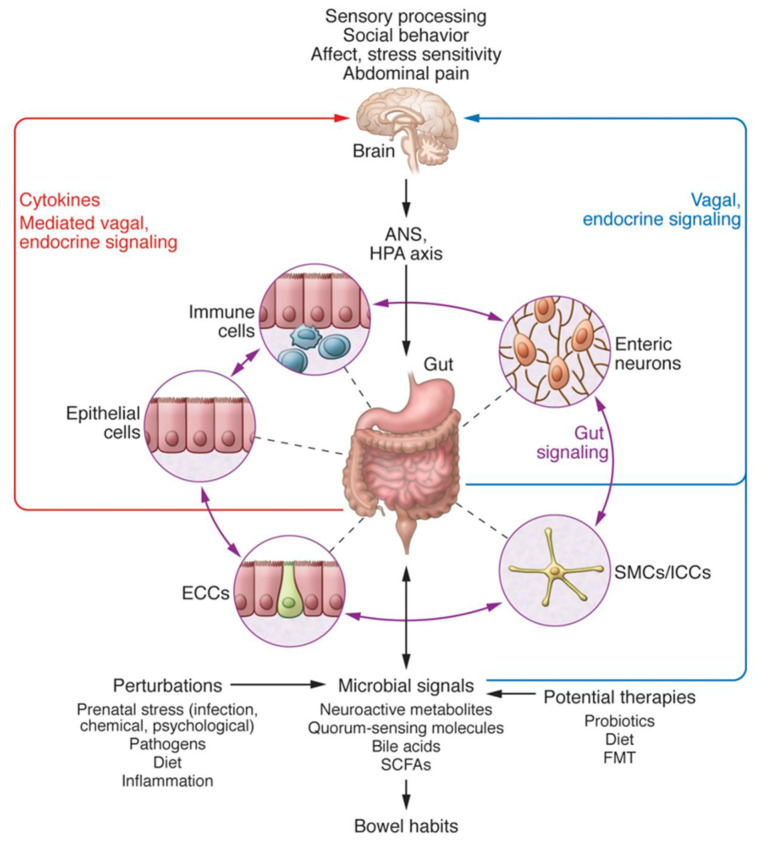
Bidirectional interactions within the Brain-Gut-Microbiome (BGM) System taken from Mayer et al. [6]. The BGM system comprises a complicated network with multiple feedback loops that allow signaling between microbiota and the brain and gut connectomes. The microbiome can modulate sensory processing, social behavior, affect, and the two arms of the stress response, in addition to abdominal pain, directly via various neuroactive and inflammatory signaling molecules, or indirectly via the vagus nerve. In turn, the brain can modulate gut microbial composition and function directly by the release of neuroactive compounds into the gut lumen acting on receptors of certain gut microbes, or via the regulation of intestinal motility and secretion activities, indirectly affecting the composition and functions of the gut microbiome. Both prenatal and postnatal perturbations to the BGM system, including but not limited to diet, infection, inflammation, and psychosocial stress, can influence the stability of these neural, neuroendocrine and immunoregulatory communication channels to create fundamental changes in brain structure and function. ANS = autonomic nervous system; HPA = hypothalamic-pituitary-adrenal; SMC = smooth muscle cells; ICC = interstitial cells of Cajal; ECC = enterochromaffin cells; SCFAs = short-chain fatty acids; FMT = fecal microbial transplant. Copyright © 2021, American Society for Clinical Investigation. The request has been put in and we are waiting for documentation.

The BGM system closely interacts with several other biological systems that regulate the body, including the immune system, hypothalamic-pituitary-adrenal axis, and the two branches of the autonomic nervous system (Figure 1) [5,6]. The afferent vagus nerve transmits information from visceral organs to brain regions, such as the hypothalamus, amygdala, and the insular cortex [7], as well as brainstem nuclei, which in turn are critical for the bidirectional communication between the gut and the brain [8]. These multiple lines of communication work in conjunction, allowing the brain and gut to influence each other [6]. Research on how the BGM system influences cognition and behavior, in particular, has been explored over the last decade; however, it is still largely in its nascent stages. 

### 3.1. Gut Microbiota and Development

**Figure 2:**
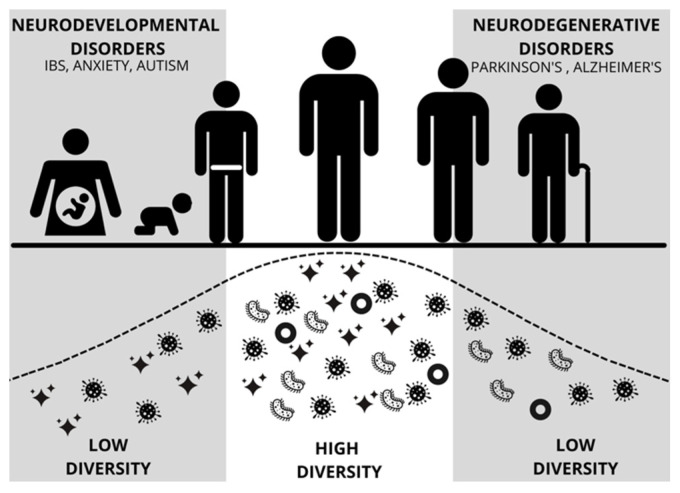
Figure modified from Mayer [9] depicting diversity and abundance of gut microbes across the lifespan of a human. Early and late periods of low diversity coincide with vulnerability to neurodevelopmental disorders and neurodegenerative disorders, respectively. IBS = Irritable Bowel Syndrome.

Gut microbiota development during the first 1000 days of life (including prenatal life) is critical for establishing a healthy and protective microbiome (Figure 2) [9]. Data indicate that one’s microbiome begins to develop rapidly following birth, with influencing factors such as the delivery method, infant feeding practices, antibiotics, and the environment [10]. Early life dysbiosis may be especially impactful in early neurodevelopment with the potential to alter the integrity of the blood-brain barrier and alter brain-gut signaling, both of which can lead to adverse health outcomes later in life [11,12]. In addition, prenatal maternal factors, including psychosocial stress, infections, obesity, and metabolic syndrome, can result in maternal dysbiosis and dysregulated maternal immune activation, posing significant risks to offspring neurodevelopment [13]. Throughout early life and childhood, microbiota continue to play major roles in modulating immune system functioning, as well as the maturation of the brain and body of the host [11,14,15,16]. 

Similarly, in early neural development, the developing microbiome appears to be particularly sensitive during early life and its profile can be altered by external stimuli, including stress, adversity, diet, environmental microbes, and antibiotics, with both immediate and long-term negative effects on the integrity of the immune system, metabolism, and overall health [17,18,19]. Such perturbations during sensitive periods of development have also been linked to negative cognitive outcomes [20,21,22], socioemotional functioning [23,24,25,26], and internalizing and externalizing problem behaviors [21,22], all of which have serious implications for various neurodevelopmental disorders.

### 3.2. Gut Microbiota and Clinical Symptomatology in ASD

Previous research has shown an association between gut microbiota and various neuropsychiatric disorders, including attention deficit hyperactivity disorder (ADHD), depression, and obsessive-compulsive disorder [27], which often co-occur with ASD [28]. ASD symptomatology has also been directly associated with gut microbiota [29]. Based on these reported associations and the multitude of potential therapeutic targets, new therapeutic interventions targeting the BGM system have been proposed and evaluated [30,31]. Below, we focus on current research that informs our understanding of gut microbiota and ASD, including GI disorders, social deficits, disruptions in neurochemical mechanisms, and abnormal brain structure and function.

## 4. Gut Microbiota and ASD Symptomatology 

### 4.1. Gut Microbiota and GI Impairment in ASD

GI symptoms—abdominal pain, constipation, and diarrhea, in particular—have been reported in 46–84% of individuals with ASD [32], which has led to the hypothesis that gut dysbiosis may be especially relevant to ASD patients with GI distress. Studies investigating the gut microbiome of children with ASD have found abnormal gut-derived metabolite patterns as well as certain taxa that significantly differ in relative abundance from healthy controls (e.g., *Clostridia*, *Desulfovibrio*, *Bifidobacteria*, *Bacteroides*) and are strongly associated with GI symptoms [33,34,35,36,37,38,39]. To date, the exact microbial composition associated with ASD has yet to be determined, with contradictory findings existing at the phylum, genus, and species levels, as well as in alpha and beta diversity; for a review, see [34]. It is important to note that this lack of consensus may be due to several factors, including study-wide differences in collection methods, preprocessing, statistical analysis, age, sex, participant diet, specimen type, ASD heterogeneity, and presence of GI disorders, all of which demonstrate a need for more homogenous samples and standardized collection and analysis procedures [40].

### 4.2. Gut Microbiota and ASD-Related Behavior

It has been theorized that a long history of coevolution has closely linked social behavior and gut microbiota across the animal kingdom [41]. A bidirectional relationship has been observed in animal models in which the gut microbiome influences social behavior, while social interactions and social structures also shape the composition and function of the microbiome [41,42,43]. Much of the work tying gut microbiota to social behavior has been conducted in rodent models [44], including germ-free rodents [45,46,47] and mouse models known for their phenotypic similarities to humans with ASD (e.g., repetitive movements, low reciprocal social interactions) [48,49]. These studies have utilized both bottom-up (e.g., manipulating the presence of bacteria by colonizing germ-free mice) [47] and top-down (e.g., starting with a genetic mouse model and investigating their gut microbiome) [48] approaches.

In experiments conducted by Desbonnet et al. [47], male mice under germ-free rearing conditions engaged in social avoidant and repetitive behaviors and displayed a lack of interest in social novelty and social motivation, all of which are considered ASD-like behaviors. When the microbiome of a second set of germ-free mice was colonized with fecal bacteria from neurotypical, normally behaving mice, many of these behavioral deficits were reversed, demonstrating the significance of healthy microbiota for typical social functioning in mouse models. These data support the notion that gut bacteria modulate ASD-like symptoms. Using a top-down approach, Golubeva and colleagues [48] investigated the interaction between GI physiology, microbiota composition, and social behavior in BTBR T+Itpr3tf/J (BTBR) mice, which are well-validated models known to exhibit ASD-like behaviors. They found that BTBR mice displayed altered relative abundance levels in 18 out of 44 identified gut bacterial genera, which have been linked to physiological and behavioral impairments. Specifically, the mice showed significantly reduced amounts of *Bifidobacteria* and *Blautia* species, both of which play vital roles in optimal GI and metabolic functioning and social interactions within the BTBR mouse strain. This finding is consistent with those of Wang et al. [50], who compared the fecal samples of children with ASD and neurotypical controls and observed reduced abundances of *Bifidobacterium* in the ASD group. Additional bacterial taxa, including *Akkermansia*, *Bacteroides*, *Desulfovibrio*, and *Lactobacillus*, were found to have abnormal abundances in the gut of BTBR mice [48]. These taxa have also been associated with ASD symptomatology, including social, repetitive, and anxious behaviors in both animal and human studies [29,35,50,51,52,53].

In both human subjects and mouse models of ASD, gut microbiota-associated metabolites have been linked to ASD symptoms and co-occurring GI abnormalities [33,34,35,36]. Needham et al. [36], however, reported significant differences between the fecal and plasma metabolomes of typically developing (TD) and ASD children regardless of the presence of GI symptoms in the ASD group, demonstrating that microbial abnormalities and their influence on behavior may not be unique to ASD patients with GI dysfunction. Interestingly, metabolite levels were correlated with clinical behaviors, as measured by two clinical ASD assessments (Autism Diagnostic Observation Schedule [ADOS] and Autism Diagnostic Interview-Revised [ADI-R]), demonstrating significant relationships between metabolites, GI function, and behavior [36]. In another notable human-mouse microbiome study, Sharon and colleagues [54] transferred gut microbiota from ASD and TD hosts into germ-free wild-type mice. After the colonization of ASD microbiota, the mice displayed various hallmark ASD-like behaviors, such as increased repetitive behavior and decreased locomotion and communication, as well as different metabolome profiles compared to offspring mice colonized with microbiota from TD controls [54]. In addition to these behaviors, more recent studies have found decreased sociability, decreased sensitivity to social odors, and dysregulated metabolic pathways and metabolites in germ-free mice colonized with ASD bacteria [36,55]. Taken together, the studies above suggest that altered gut microbiota and some of their metabolites influence ASD-like behaviors in rodent models and ASD symptoms in patients. 

### 4.3. Gut Microbiota Therapy and the Reduction of ASD Symptomatology

#### 4.3.1. Probiotic Therapy

**Figure 3:**
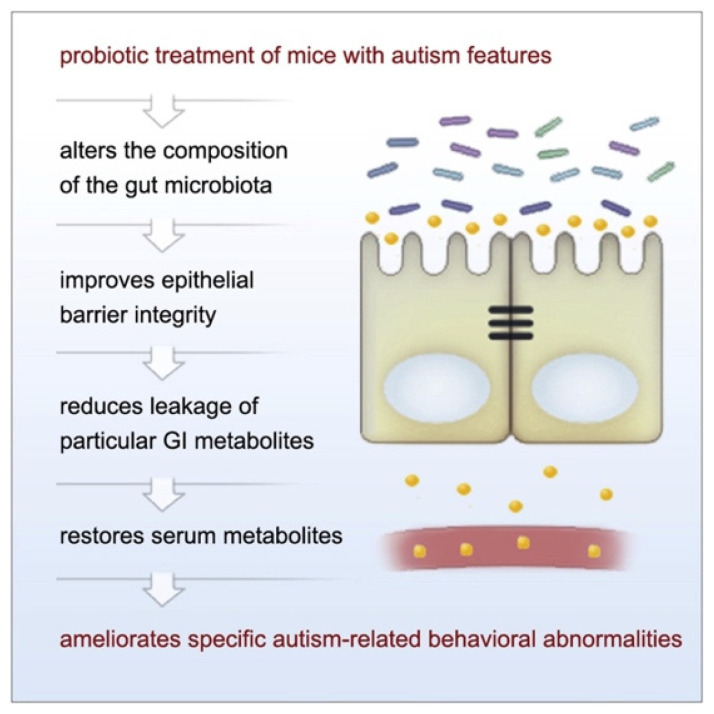
The observed relationship between probiotic use and amelioration of ASD-like symptoms in MIA offspring. Figure taken from Hsiao et al. [49] Graphical Abstract. Cell 2013 155, 1451–1463 DOI: (https://doi.org/10.1016/j.cell.2013.11.024) Copyright © 2021 Elsevier Inc.

Given the prior findings on microbiota and behavioral symptoms in ASD, a number of studies have investigated the use of probiotics (“live microorganisms which when administered in adequate amounts confer a health benefit on the host” [56]) as a potential treatment. Indeed, probiotics can alleviate GI symptoms in both ASD and TD populations [57,58,59,60]. Thus, the question of whether probiotics can also be used to treat behavioral symptoms of ASD has been explored, particularly in rodent models. Hsiao et al. [49] used a maternal immune activation (MIA) mouse model whose offspring display many core phenotypes of ASD (e.g., altered communication, abnormalities in social behavior, stereotyped behaviors) following prenatal maternal treatment with the viral polyinosinic-polycytidylic acid (poly I:C) during specific points of their neurodevelopment [61]. It was observed that MIA offspring displayed ASD-related behavioral abnormalities while also having increased intestinal permeability, microbiome changes, and metabolomic alterations [49]. Oral administration of the gut commensal *Bacteroides fragilis* corrected gut permeability, improved gut microbiota and blood metabolite profiles, and ameliorated atypical anxiety, communicative (i.e., ultrasonic vocalizations), repetitive, and sensorimotor behavioral symptoms, but not sociability or social preference, in the MIA offspring (Figure 3) [49].

Although the amelioration of all social symptoms was not observed in the aforementioned study, preclinical evidence suggests that other strains or types of microbes may improve social behavior [49]. In fact, several studies have demonstrated that various strains of *Lactobacillus* reduce social impairments in animal models [44]. Buffington et al. [45], for example, found that introducing *L. reuteri* reduced social deficits in maternal high-fat-diet rodent offspring, which are born with gut-microbial alterations detrimental to their social functioning. In support of using *Lactobacillus* strains as a means for treating ASD symptoms, a more recent study demonstrated that a *L. plantarum* PS128 intervention for children with ASD reduced aberrant behaviors commonly seen in individuals with ASD and may ameliorate social communicative impairments [62].

#### 4.3.2. Fecal Microbiota Transplantation Therapy

Fecal microbial transplantation (FMT) has been shown to alleviate the behavioral symptoms of various neuropsychiatric disorders, including ASD [63]. A recent exploratory, unblinded, and non-randomized clinical trial, involving 18 children diagnosed with ASD (with moderate to severe GI issues) and 20 TD children matched by age and gender without GI disorders, evaluated the effect of repeated Microbiota Transfer Therapy (MTT), a modified FMT, on gut microbiota composition and GI and ASD-related symptoms [64]. MTT, which combined antibiotic treatment, a bowel cleanse, a stomach-acid suppressant, and an extended fecal microbiota transplant, led to significant improvements in both GI and ASD symptoms, including improvements in social skill deficits [64]. A majority of these changes were sustained and even improved two years after completion of the treatment [65]. Additionally, it was shown that both the plasma and fecal metabolite profiles of the ASD group became more similar to those of their TD counterparts following MTT [66,67]. The effects of FMT have also been evaluated in adult pathogen-free mice using donor and in vitro-cultured human gut microbiota. Chen et al. [68], for example, reported significant improvements in behavioral impairments associated with ASD, particularly for anxiety-related and repetitive behaviors, as well as moderate improvements in social behavior, with the findings in alignment with previous literature. Taken together, data from rodent models and preliminary clinical studies suggest that interventions involving both probiotics and FMT may offer promising lines of research for understanding ASD and may help in the development of novel therapies.

## 5. Putative Mechanisms of the BGM System Related to ASD

**Figure 4:**
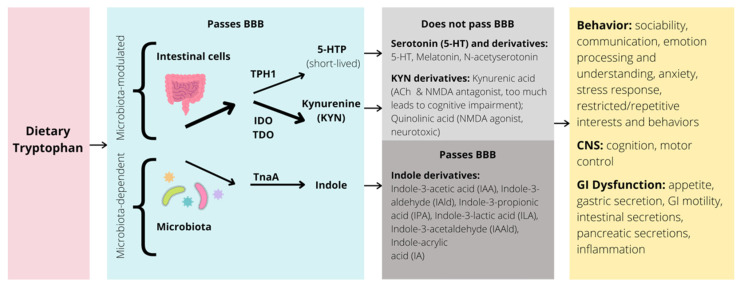
Simplified illustration of the tryptophan pathway in the GI tract and potential impacts on behavior, CNS, and GI dysfunction. BBB = blood brain barrier; TPH = tryptophan hydroxylase; IDO = indolamine 2,3-dioxygenase; TDO = tryptophan 2,3-dioxygenase; TnaA = tryptophanase; 5-HTP = 5-hydroxytryptophan; 5-HT = 5-hydroxytryptamine, or serotonin; ACh = acetylcholine; NMDA = N-methyl-D-aspartate; CNS = central nervous system; GI = gastrointestinal; arrow thickness represents strength of pathway.

Several models have been put forth hypothesizing biological mechanisms associated with abnormal gut microbiota and symptoms in ASD. One mechanism relevant to modulating the clinical symptoms of ASD is the metabolism of the essential amino acid tryptophan along the BGM system. Here, we discuss the influence of gut microbiota on the tryptophan metabolic pathways in both animals and humans, as well as how disruptions to these pathways influence ASD social and behavioral deficits (Figure 4). 

### 5.1. Tryptophan Pathways

Dietary tryptophan is metabolized through three main pathways: the serotonin, kynurenine (KYN), and indole metabolic pathways. Over 95% of tryptophan is oxidized and degraded to yield metabolites along the KYN pathway [69]. Importantly, tryptophan is also the sole precursor to the neurotransmitter serotonin (5-HT) in the brain and gut (synthesized by action of enzyme tryptophan hydroxylase [TPH]) [69]. Whereas gut microbiota play a modulatory role in the balance between serotonin and KYN production, the biosynthesis of indoles and indole derivatives (e.g., indole-3-aldehyde, indole-3-acetic acid, indole-3-propionic acid) from tryptophan is fully dependent on the enzyme tryptophanase, only found in select microbes [70]. How changes in the relative abundance of certain gut microbiota contribute to modifications of these pathways, central tryptophan metabolism, and ultimately brain function and behavior, is a crucial and ongoing area of research [71]. Many of these findings also require analysis of how this relationship modulates clinical symptoms characteristic of neurodevelopmental disorders, such as ASD.

#### 5.1.1. Indole Pathway and ASD

Indole synthesis is driven by certain bacterial taxa that convert undigested tryptophan from the gut lumen into indole and indole derivatives, constituting an exclusively microbe-dependent pathway [70]. Many of the phyla, genera, and species associated with the production of indoles and altered indole products involved in tryptophan metabolism have been linked to the development of ASD and related neuropsychiatric disorders [33,55,72,73,74,75]. Mice from the MIA model have shown abnormally high levels of key serum metabolites produced by gut microbes, including 4-ethylphenylsulfate, serum indolepyruvate, and indole-3-acryloylglycine, all of which were readjusted by treatment with *B. fragilis* [49]. In comparison, a human study also found that urinary metabolites of ASD and TD children significantly differed along the tryptophan and purine metabolic pathways, suggesting that the gut microbiome contributes to abnormal tryptophan metabolism in ASD [73]. Specifically, gut bacteria-derived metabolites indolyl-3-acetic acid and indolyl-lactate were more numerous in the ASD group compared to controls [73], consistent with the findings from Xiao et al. [55] in the cecal matter of mice that had received FMT from ASD donors. These altered pathways overlapped with those of rodent models which displayed ASD-like behaviors, demonstrating a potential pathophysiological explanation for many behavioral symptoms of ASD [73]. Similarly, De Angelis et al. [72] found increased indole and 3-methylindole in the fecal samples of ASD children. After indole is absorbed in the gut, it is oxidized and sulfated by liver enzymes into indoxyl and indoxyl sulfate metabolites, respectively. Interestingly, these indole metabolites have been identified as potential metabolic markers for ASD, as well [76,77].

#### 5.1.2. Kynurenine Pathway and ASD

The KYN pathway, also derived from tryptophan and modulated by gut microbes, largely depends on indoleamine-2,3-dioxygenase (IDO) and, to a lesser degree, tryptophan-2, 3-dioxygenase (TDO) for metabolization [78]. IDO, expressed in all body tissues, is typically activated in the presence of pro-inflammatory cytokines, whereas TDO, expressed primarily in liver tissues, is activated by glucocorticoids [78,79]. Once transformed from tryptophan, KYN metabolizes into two downstream metabolites, neuroprotective kynurenic acid (KA) and neurotoxic quinolinic acid (QA) [78]. Recent evidence suggests that altered KYN metabolism is indicative of greater tryptophan depletion and an impaired serotonergic pathway in ASD [80]. In a study investigating the role of the KYN pathway in ASD, Bryn et al. [81] showed that children with ASD had significantly lower KA serum levels, higher KYN/KA ratios, and higher QA serum concentrations than TD children. These findings are consistent with those of Gevi et al. [73], who found that tryptophan was disproportionately metabolized into QA, with significantly decreased levels of KA, in children with ASD. Both studies demonstrate an increased potential for neurotoxicity in children with ASD, which is thought to be involved in the pathophysiology of the disorder [73,81]. Interestingly, Xiao et al. [55] found increased KA in mice following FMT from children with ASD. These levels correlated with specific bacteria (e.g., genera in the orders *Clostridiales* and *Bacteroidetes*), supporting their modulatory role in tryptophan metabolism, but demonstrating a need for further research on how microbiota alter KYN-pathway products [55].

Although there is minimal literature associating gut microbiota with the KYN pathway in human ASD populations, studies in other clinical and normative populations have provided evidence to support this relationship [78]. Interestingly, Luna et al. [82] found in their study of ASD microbiome-neuroimmune signatures that along with tryptophan and serotonin levels, inflammatory cytokine levels correlated with certain bacterial species in children with ASD and functional GI disorder comorbidities. No direct link was made to the KYN pathway; however, because IDO is typically activated in response to cytokines [78,79], there is reason to investigate whether the abnormal microbial profile of individuals with ASD may be implicated in the dysregulation of the KYN pathway.

#### 5.1.3. Serotonin Pathway and ASD

Serotonin (also referred to as 5-HT) is important for mood regulation, higher order cognition, and neurodevelopment of both the CNS and ENS [83,84]. Although the majority (>90%) of serotonin comes from enterochromaffin cells in the GI tract, serotonin is also synthesized in the neurons of the ENS and CNS, particularly the raphe nuclei in the brainstem [85]. Gut microbiota and their metabolites can influence central and peripheral serotonin production and metabolism through a variety of mechanisms [71,86]. Because only a small percentage of tryptophan is converted into serotonin, any alterations to its metabolism and availability can pose a significant risk to one’s health [87].

Approximately 30% of ASD patients have hyperserotonemia, or elevated whole-blood serotonin levels [88], which is believed to be due in part to increased serotonin production in enterochromaffin cells in the gut [89]. Based on similar and replicated findings, it has been postulated that hyperserotonemia may represent a highly heritable biomarker of ASD and that the serotonin pathway as a whole may be dysfunctional in at least a subgroup of ASD individuals [89,90]. In preclinical models, hyperserotonemia has been linked to social-behavioral deficits characteristic of ASD [49,91,92]. Tanaka et al. [92], for example, found that a tryptophan-depleted diet, which decreases brain serotonin levels and regulates gene expression inside the serotonin system, improved social impairments of genetically modified ASD mouse models. Lim et al.’s [91] report of elevated serum serotonin levels in environmental risk factor mouse models of ASD that were associated with changes in bacteria known to stimulate serotonin production suggests that alterations in serotonin and hyperserotonemia itself may have a microbial origin. The connection between serotonin and the microbiome has been made in humans as well, as demonstrated by a link between increased GI symptom severity and hyperserotonemia in ASD youth [93]. Other studies investigating serotonin-related dysfunction in children with ASD and co-occurring GI symptoms have implicated fecal metabolites in the metabolic network of various neurotransmitters, including serotonin [33], and have found increased levels of serotonergic metabolites, including 5-HIAA, the main metabolite of serotonin, in the rectal tissue of ASD youth with co-occurring functional GI disorders [82]. These metabolite levels correlated with the dysbiosis of several bacterial species, demonstrating a potential microbiome profile for ASD [82].

SERT Ala56, the most common variant of the serotonin-selective transporter responsible for serotonin reuptake in both the brain and intestines, has been found to be overexpressed in ASD patients and linked to neurobiological and GI symptoms in a genetically modified murine ASD model [94]. SERT Ala56 mice are also known to exhibit serotonin-related dysfunction, including excess clearance of central serotonin, augmented serotonin receptor sensitivity, and hyperserotonemia [95]. Research supporting connections between an altered serotonin system and ASD pathophysiology has demonstrated a positive relationship of serotonin and SERT levels with autism symptom severity in humans [96]. Furthermore, numerous animal studies have implicated gene polymorphisms of SERT, as well as genetic and surface transporter expression and function, in the underlying repetitive behaviors and social behavior deficits of ASD, for a review, see [97].

Contributing to the link between serotonin, gut microbiota, and ASD, the BTBR inbred strain has been shown to display (1) reduced SERT density and binding throughout the brain and increased serotonin activity in the hippocampus (a brain region involved in learning, social, and emotional processing, and found to be abnormal in ASD) [98,99,100]; (2) changes in intestinal microbiota associated with slowed GI motility and impaired intestinal serotonin production [48]; and (3) increased sociability following brief exposure to serotonin reuptake inhibitors [98,101] and tryptophan supplementation [102]. Taken together, these studies support the hypothesis that altered gut microbiota are involved in the tryptophan-serotonin metabolic pathway in ASD and provide a framework for future studies aiming to alleviate the GI and, consequently, behavioral symptoms of ASD patients.

A recent study by Fung et al. [103] showed that the gut bacterium *Turicibacter sanguinis* expresses a neurotransmitter sodium symporter-related protein with sequence and structural homology to mammalian SERT. This microbe imports serotonin through a mechanism that, like its host homologue, is inhibited by the selective serotonin reuptake inhibitor, fluoxetine. Serotonin reduces expression of sporulation factors and membrane transporters in *T. sanguinis*, which is reversed by fluoxetine exposure. Treating *T. sanguinis* with serotonin or fluoxetine modulates its competitive colonization in the GI tract of antibiotic-treated mice. In addition, fluoxetine reduces membership of *T. sanguinis* in the gut microbiota of conventionally colonized mice. One may speculate that genetic variants exist for the microbial SERT-like mechanism and that alterations in the bidirectional host-microbe interactions in tryptophan metabolites play a role in ASD pathophysiology, including gut symptoms.

### 5.2. Serotonin in the Brain and Relationships with Behavior

Several functional neuroimaging studies indicate that the microbiota-modulated serotonergic system affects neural functioning in ASD. For example, positron emission tomography (PET) ligand studies have observed atypical serotonin functioning throughout the brain that was associated with greater social deficits in individuals with ASD [104,105]. One study found that ASD participants had significantly less binding of thalamic serotonin receptors than age-matched controls and that binding potential in the ASD group was negatively associated with social communication impairments [104]. Another PET study found a global reduction in SERT binding in adults with ASD [103]. Moreover, reduced SERT binding in the anterior and posterior cingulate cortices (ACC; PCC) was associated with impaired social cognition and reduced binding in the thalamus was associated with repetitive behaviors [105]. A more recent PET study found lower serotonin transporter availability in the total gray matter and brainstem of adults with ASD, relative to matched controls [106]. Serotonin transporter availability in the nucleus accumbens, ACC, and putamen was positively correlated with social cognition, indicating that the serotonin transporter may be a marker that can be targeted in pharmacological interventions [106].

Additionally, a polymorphism of the serotonin transporter gene (serotonin-transporter-linked-promoter region; 5-HTTLPR) has been shown to affect brain function in ASD. Short variants of 5-HTTLPR, which reduce serotonin transporter expression [107], are associated with impairments in social communication and interactions in individuals with ASD [108,109]. This is in line with other findings that have linked 5-HTTLPR to the default mode network (DMN), a neural network engaged during passive self-referential cognitive processing and associated with social cognition [110]. Wiggins et al. [111] found that while youth with ASD who had low expressing 5-HTTLPR alleles had stronger posterior-anterior DMN connectivity than those with high expressing genotypes, the opposite was true for TD controls. Thus, resting-state connectivity was shown to be influenced by 5-HTTLPR in a different pattern in ASD than typical controls, suggesting that individuals with low expressing 5-HTTLPR genotypes may comprise a subtype of ASD.

The same researchers showed that ASD youth with low expressing 5-HTTLPR genotypes also had atypical amygdala functioning while performing social tasks [112,113]. During an observational face-processing task, youth with low expressing 5-HTTLPR genotypes failed to show amygdala habituation to repeated observation of sad faces compared to TD controls and ASD youth with high expressing genotypes. Building on these findings, Velasquez et al. [112] showed that when viewing happy faces, the ASD group with the low expressing genotypes had abnormally high rates of functional connectivity between the amygdala and subgenual ACC than ASD individuals with higher expressing genotypes and TD groups of higher and lower expressing genotypes. ASD participants with greater amygdala-subgenual ACC connectivity, key regions involved in emotion arousal and regulation, also showed higher expressing genotypes of 5-HTTLPR and less social dysfunction. Together, these findings indicate that, in ASD, it is possible that some socioemotional functioning during rest and social processing may be influenced by atypicalities in the serotonergic system [112,113]. These results further support the importance of the 5-HTTLPR genotype in examining the heterogeneity of social function in ASD. Nevertheless, a recent meta-analysis found no support for a direct effect of 5-HTTLPR polymorphism on risk of ASD, indicating that patterns across studies may be difficult to find given the heterogeneity of the disorder. Further analyses in larger sample sizes with more homogeneous subgroups of ASD participants may be necessary [114].

Research also suggests that there are group differences between ASD and TD neural responses to serotonin availability. One fMRI study found that increased serotonin (via a selective serotonin reuptake inhibitor) was associated with sustained neural activation in emotion-related brain regions during a negative facial emotion-processing task in ASD adults compared to neurotypical controls, which exhibited an expected habituation response [115]. The authors concluded that homoeostatic control of these regions is altered by serotonin in ASD. Two other fMRI studies found that acute tryptophan depletion (which lowers serotonin levels) was associated with deficits in facial affect [116] and inhibitory processing [117] in ASD. These findings may help explain some of the social and restricted/repetitive behaviors that are hallmarks of the disorder [116,117]. Further, many of the brain regions found to be modulated by the tryptophan depletion have previously been reported to have serotonergic abnormalities in people with ASD (e.g., synthesis differences, lower numbers of receptors and transporters) [116].

Despite the extensive evidence showing alterations in the tryptophan pathway and the serotonergic system in ASD, there is no consensus in current literature on tryptophan availability and its influence on behavior in patients with ASD. While studies have suggested that reduced levels of tryptophan [118,119,120] and decreased tryptophan metabolism are prominent features of ASD [121], a recent study showed both increased and decreased levels of tryptophan in ASD youth [122], which, again, may be related to ASD heterogeneity and marks the need for ASD sub-grouping. The potential roles of oxytocin and other upstream markers in ASD have also been discussed [123] and it is likely that interactions between oxytocin and serotonin may further influence the pathophysiological processes of ASD [124]. More research is necessary to fully understand the complex neurochemical processes underlying the relationship between the BGM system, tryptophan metabolism, and ASD. Below, we discuss specific findings in BGM connections at the structural and functional levels and how changes in the microbiome impact neural networks.

## 6. Microbiota and the Social Brain: Structure and Function

Preclinical studies showing an interplay between gut microbiota and social behavior emphasize the role of microbiota on brain function. Recent studies combining neuroimaging with gut microbial profiling in humans have highlighted the relationship between gut microbiota and the structure and function of brain regions and networks known to be altered in ASD; for a review, see [125,126]. To our knowledge, no studies to date have looked at gut microbiota and neural functioning in individuals with ASD. Nevertheless, a growing range of observations suggests that brain function may be modulated by changes in the gut microbiome via metabolic and signaling pathways. In particular, the amygdala, cingulate, and insula—which are important for social cognition and emotional regulation—receive afferent visceral input and are known to have abnormal processing in ASD [127,128,129,130,131,132,133,134,135,136,137,138]. These regions are also a part of larger neural networks involving salience and socioemotional processing, which are also thought to be altered in ASD [139,140,141,142,143,144]. How gut microbiota is related to these regions in ASD is still unknown, but recent research in various populations suggests there may be a connection.

### 6.1. Amygdala and Microbiota

The amygdalae are bilateral nuclei primarily involved in high-level information processing, emotional processes and behaviors (e.g., fear, aggression), decision making, and social interaction [145]. Newly emerging research has begun to focus specifically on how gut microbiota modulate amygdala function and the possible implications for treating psychiatric disorders associated with amygdala dysregulation, including the reduction of symptom severity in ASD [146]. Numerous genetic, epigenetic, and environmental changes in early development have been linked to gut microbial alterations, which may in turn alter amygdala development and white matter connections to other brain regions; for a review, see [147]. Rodent studies have found microbial status to influence amygdala volume, dendritic morphology, and spine density [148], as well as gene expression and neural development of the amygdala during critical time windows [149,150]. Another recent study demonstrated that the presence of microbiota is necessary for socially induced gene expression regulation in the amygdalae [151]. Moreover, Lobzhanidze et al. [152] recently showed that the microbial metabolite propionic acid, which in excess has been associated with ASD symptomatology [153,154], significantly reduces social motivation and alters amygdalae structure in rodents. At the functional level, microbiome diversity has been negatively associated with functional connectivity of the amygdala during early brain development in humans [155]. Since the developmental trajectory of the functional connectivity of amygdalae during the first two years of life has been shown to be a significant predictor of cognitive and emotional outcomes [155,156], the potential impact of gut microbiota on emotion-related brain region connectivity and growth patterns in an early critical period may have long-term implications for mental health, including ASD symptomatology.

Given the strong relationship between the amygdala’s impairments in ASD [157,158,159,160] and current research demonstrating gut microbiota’s relationship to the amygdala, understanding how gut microbiota interact with amygdala structure and function in ASD may elucidate underlying neural correlates of social and emotional processing deficits. Further research examining sociality and gut microbiota in individuals with ASD may help inform treatment targeting amygdala-dependent behaviors related to ASD via the BGM system.

### 6.2. Insula and Microbiota

Subregions of the insular cortex play important roles in the processing of sensory, autonomic, and interoceptive information from the viscera and are thought to be involved in socioemotional processing [161]. The anterior insula, specifically, serves as a crucial hub of a network involved in detecting salient events and integrating internal physiological signals (including gut signals) [162] and external sensory stimuli to guide behavior [163]. Reduced connectivity in the anterior insula and dysfunction of this network have been consistently implicated in ASD [138]. It has also been proposed that modulations to this network may play an important role in regulating immune function and GI symptoms in IBS [164].

To date, no studies have examined the relationship between the insula and the microbiome in individuals with ASD; however, there is evidence from healthy individuals and those with irritable bowel syndrome (IBS) to suggest that insular structure is indeed modulated by changes in the microbiome, which contribute to the pathophysiology of IBS [165,166]. Structurally, cortical thickness of the insula has been found to be related to both IBS [167,168,169] and microbiota composition. Tillisch et al. [170] found a positive association between cortical thickness in the insula and *Bacteroides* abundance in healthy adult women. The same group investigated the gut microbial composition and structural brain signatures of individuals with IBS and discovered a greater volume in the mid and posterior insula in IBS patients, relative to healthy controls [171]. In a subgroup of patients with a distinct microbial makeup, Labus et al. [171] found that the anterior insula had a larger surface area and smaller cortical thickness relative to healthy controls. Additionally, functional alterations of the insula have been reported by several fMRI studies in patients with IBS [172,173,174,175,176].

In a recent study directly investigating the relationship between gut microbiota and neuronal activity in the insula, Curtis and colleagues [177] observed that microbiota diversity was positively associated with resting-state functional connectivity between the middle insular cortices and frontal and cerebellar areas. Furthermore, when controlling for smoking status (previously shown to influence gut microbiota [178]), the connections between anterior and inferior regions of the insula and various brain regions were also related to microbiota diversity. Notably, they reported correlations between anterior and inferior insula connectivity and *Bacteroides* and *Prevotella*, two genera with well-established abnormalities in individuals with ASD [53,179,180]. Moreover, levels of fecal microbiota-derived indole metabolites (i.e., indole, skatole, indoleacetic acid) have been positively associated with the anatomical and functional connectivity of the amygdala-anterior insula circuit in healthy individuals [181]. Taken together, these findings indicate that microbiome differences in individuals can be observed at the cortical level. Thus, there is a need for research on how microbiome diversity and regulation may influence brain activity and help treat neuropsychiatric disorders associated with insular abnormalities, including ASD.

### 6.3. Gut Microbiota and Other Emotion-Related Brain Regions

Several neuroimaging studies have reported strong relationships between gut microbiota and functioning of emotion-related brain regions, in addition to the amygdala and insula. For example, in an fMRI study utilizing an emotional faces attention task, consumption of a fermented milk product with probiotic was related to reductions in activity of a widely distributed functional network containing brain areas that control processing of emotion, sensation, attention, and interoception in healthy women [182]. In addition to the insula and amygdala, this network included a range of midbrain regions centered on the periaqueductal gray [182]. A similar connection was suggested when treatment with *Bifidobacterium* was found to be associated with both decreased depression and reduced activity in the amygdala and fronto-limbic regions in IBS patients [183].

More recently, Gao and colleagues [155] investigated the microbiome’s potential relationship with emotion-related brain circuits in infants through fecal analyses and resting-state fMRI data. Strong negative associations were found among alpha diversity and connectivity between the amygdala and thalamus as well as the right anterior insula and ACC, suggesting that higher levels of microbiome diversity may be related to less efficient mechanisms for emotion and threat processing. The researchers also observed a positive correlation between alpha diversity and sensorimotor-parietal connectivity, which allows for interactions between auditory, visual, and somatosensory cortices and has been linked to cognitive ability at two years of age [155]. These results provide initial evidence for microbiome-associated changes in functional neural circuits during early human development [155]. Future studies may explore the relationships between connectivity and ASD-related symptomatology, particularly the emotion and sensory processing atypicalities common in individuals with ASD [184,185].

Microbiota have also been related to white matter microstructure and cortical thickness in other emotion- and cognition-related brain regions. One study on obese and non-obese participants found significant positive associations between fecal microbiota diversity and fractional anisotropy (FA; a measure of white matter connectivity) of the hypothalamus, caudate nucleus, and hippocampus [186]. Additionally, abundance of *Actinobacteria* was positively associated with executive functioning and structural microstructure, as measured by FA in the amygdala and thalamus, supporting the link between the microbiome and emotion processing [186]. Tillisch and colleagues [170] found further evidence that gut microbiota may impact brain structure in addition to functional connectivity, mood, and behavior. Specifically, greater *Prevotella* abundance was related to increased connections between emotion-, attention-, and sensory-processing brain regions and lower cortical thickness in the anterior insula, while higher *Bacteroides* was associated with greater cortical thickness in the frontal cortex and anterior insula [170].

In summary, evidence from neuroimaging studies indicates that gut microbial parameters may be associated with social and emotional brain structure and function across a variety of populations, especially individuals with IBS. Researchers have also begun to use the functional brain-gut approach to gain a better understanding of neuropsychiatric disorders, including ADHD, anxiety, depression, and schizophrenia [183,187,188,189]. Given the increased prevalence of IBS and GI-related comorbidities in individuals with ASD, studies that explicitly investigate how gut microbiota are related to neural functioning in ASD are warranted.

## 7. Conclusions and Future Directions

A growing body of evidence has demonstrated associations between gut microbiota and ASD symptoms, including socioemotional behavior and GI symptoms, through various pathways within the immune, neuroendocrine, and metabolic systems. The interconnections within the BGM system are also demonstrated in neuroimaging research, highlighting the relationships between microbiota and the structure and function of brain regions (e.g., the amygdala and insula) implicated in various theories of ASD. When viewed together with findings from rodent studies, a causal role of microbiota in ASD is plausible, especially in a subgroup of patients with GI difficulties. Additional research, however, is needed to characterize the neural circuitry and mechanisms that underlie this connection. A more comprehensive understanding of the BGM interactions in ASD can prepare the way for early biomarkers of ASD and microbe-based therapeutic treatments such as targeted prebiotics, probiotics, and fecal transplants. Given the heterogeneity of ASD, we acknowledge it is unlikely that all cases of ASD are impacted by alterations to the BGM system to the same degree; identification of potential subgroups or clinical phenotypes of ASD with gut-microbial disturbances, however, can contribute to individualized medicine and therapy.

To better understand the relationship between ASD and the gut microbiome, research should be prioritized that looks at the prenatal trajectory of BGM interactions in the pregnant mother and its influence on the developing fetus. Such studies need to address what role maternal factors (e.g., inflammatory or microbial neuroactive signals in utero) play in shaping these interactions, and what consequences these maternal influences have on fetal brain development. Maternal immune activation has been shown to increase ASD risk, as seen in the offspring of the MIA mouse model [190,191], as well as in epidemiological studies [191,192,193]. Furthermore, both a maternal high-fat diet and metabolic syndrome, a disorder characterized by systemic immune activation, have been associated with an increased risk of ASD in offspring [45,194,195].

More human studies directly comparing microbiota in ASD and TD populations and exploring the causal relationships between the microbiome, behavior, and brain functioning are also needed. For example, to understand how the gut microbiome is related to social functioning, a multimodal approach might examine variations of the transcriptome and metabolome in ASD and their relationship to behavioral and neural functioning. As noted by Yap et al. [196], is important to test the mediating effects of detailed dietary data in such work to assess the microbiome’s contributions to ASD development. To understand longitudinal changes in microbial profiles in ASD, research should track how microbiota composition and metabolic states vary prenatally in mothers and across early development in both children at risk for ASD and their TD peers. By tracking microbiota in high-risk infants, specific biomarkers, such as tryptophan metabolites and inflammatory markers, may be identified. Back translational approaches using humanized gnotobiotic mouse models provide a powerful approach to validating findings from human studies in clinically relevant experimental models [197]. Further, microbiota-based intervention studies are needed to assess causality of ASD.

To date, most studies have examined the gut microbial composition in isolation using a single omics approach. A deeper understanding of the role of the gut microbiome in ASD can be achieved by understanding its interactions with host and environmental factors. Integrative multi-omics analysis approaches combining longitudinal microbiome, genomic, transcriptomic, proteomics, and metabolomic profiling with accurate dietary assessment, deep clinical phenotyping, and brain imaging are necessary to understand the complex interactions contributing to pathophysiology and symptom generation in ASD.

## Data Availability

There is no data associated with this study as it is a review article.
